# Osteomyelitis Secondary to Ponseti Method for the Treatment of Clubfoot Associated with Meningomyelocele

**DOI:** 10.7759/cureus.4301

**Published:** 2019-03-22

**Authors:** Zainab Majid, Faryal Tahir, Laila Tul Qadar, Kashif H Qadri, Sarrah Ali Asghar

**Affiliations:** 1 Internal Medicine, Dow University of Health Sciences, Karachi, PAK; 2 Pediatrics, Dow University of Health Sciences, Karachi, PAK

**Keywords:** osteomyelitis, ponseti method, congenital talipes equinovarus, meningomyelocele, clubfoot

## Abstract

Congenital talipes equinovarus (CTEV), otherwise known as clubfoot (CF), is a common congenital abnormality of the foot, stemming in most cases from an idiopathic cause or in the remaining non-idiopathic occurrences, from meningomyelocele (MMC). Ponseti method (PM), being a gold standard treatment for the correction of this foot deformity, requires a series of regular casting usually accompanied by percutaneous tenotomy of the Achilles tendon and later maintained via abduction braces. Osteomyelitis (OM), as a complication of PM, is rarely reported especially in cases of CF-associated with MMC in which majority of the patients present with varying level of sensory deficit in the lower limbs. Therefore, the absence of pain due to neuropathy leads to a delayed diagnosis of cellulitis and chronic abscesses. We present a case of an eight-year-old male child with exogenous OM as a complication of PM affecting the tarsal bones of his right foot. After an established diagnosis using laboratory results and imaging modalities, the patient was successfully treated with broad spectrum antibiotics achieving full resolution of his clinical symptoms. We report this case due to its rarity.

## Introduction

Congenital talipes equinovarus (CTEV), otherwise known as clubfoot (CF), is a common congenital abnormality of the foot, stemming in most cases from an idiopathic cause [1] or in the remaining non-idiopathic occurrences, from meningomyelocele (MMC). MMC is a frequent yet severe type of spina bifida resulting from defective closure of the spinal canal, thereby causing the corresponding vertebrae to lack neural arches that ultimately leads to the typical complications of urinary and fecal incontinence, paralysis of lower limbs and reduced sensitivity [2]. The interrelation of the complications of CTEV with MMC is strong, as evident from the statistics showing 30%-50% association [3]. However, there exists a well-established gold standard initial therapy for teratogenic CF known as the ponseti method (PM). This method for the correction of foot deformity requires a series of regular casting usually accompanied by percutaneous tenotomy of the achilles tendon, later maintained via abduction braces [4]. Although the PM holds a high success rate, few studies have reported the incidence of rare complications including loosening of cast and skin irritation, both with a frequency of 5.48% and that of infection in 2.73% cases [5]. However, osteomyelitis (OM) as a complication of PM remains unreported. Acute OM in children occurs most commonly via hematogenous spread from a distinct focus, effecting in majority cases the metaphyseal plates of long bones [6]. Contrary to this, we present a case of an eight-year-old male child with exogenous OM, which occurred as a complication of PM, affecting the tarsal bones of his right foot.

## Case presentation

An eight-year-old male child presented to the pediatric department of Dr. Ruth KM Pfau, Civil Hospital Karachi (CHK) in January 2019 with the complain of ulcer on his right foot, high-grade intermittent fever without chills, and rigors and swelling in the same foot for the past three years, one month, and one week, respectively. He also had a history of urine dribbling and physical delayed development. He was a known case of MMC that was reconstructed at one month of age. As a congenital abnormality, he was also born with CF deformity for which PM was started as a treatment at four years of age. After the removal of the first cast which was applied for six months, his mother noticed ulcer on the right foot which was spreading but went untreated.

On examination (O/E), the patient was found alert and active, lying comfortably in bed. His heart rate (HR) was 88 beats/min, blood pressure (BP) was 110/80, respiratory rate (RR) was 26 breaths/min, and he was febrile with 103°F body temperature. Upon evaluation of the right foot, we found local non-tender edema over the dorsum along with ulcer and sinuses discharging pus with palpable posterior tibial artery. A scar mark was present on his back which was due to MMC repair. Upon central nervous system (CNS) examination, motor system evaluation of lower limbs revealed increased tone, slightly exaggerated reflexes especially increased dorsiflexion with the knee flexed accompanied by clonus and a power of 5/5. All other systems were unremarkable.

Laboratory investigations revealed hemoglobin (Hb) of 7.7 gm/dl, mean corpuscular volume (MCV) of 67.9 fl (Normal [N] = 76-96), mean corpuscular hemoglobin concentration (MCHC) of 28.2 gm/dl (N = 32-36), total leukocyte count (TLC) of 19.2 x 103/μL (N = 4-11) and platelet count (PLT) of 468 x 103/μL (N = 150-400). The level of C-reactive protein (CRP) was found to be 269.1 mg/L (N = <5). The clotting profile showed an international normalized ratio (INR) of 1.09 and prothrombin time (PT) of 11.4 seconds. Blood glucose, liver function tests, blood urea, and serum creatinine were not remarkable.

**Figure 1:**
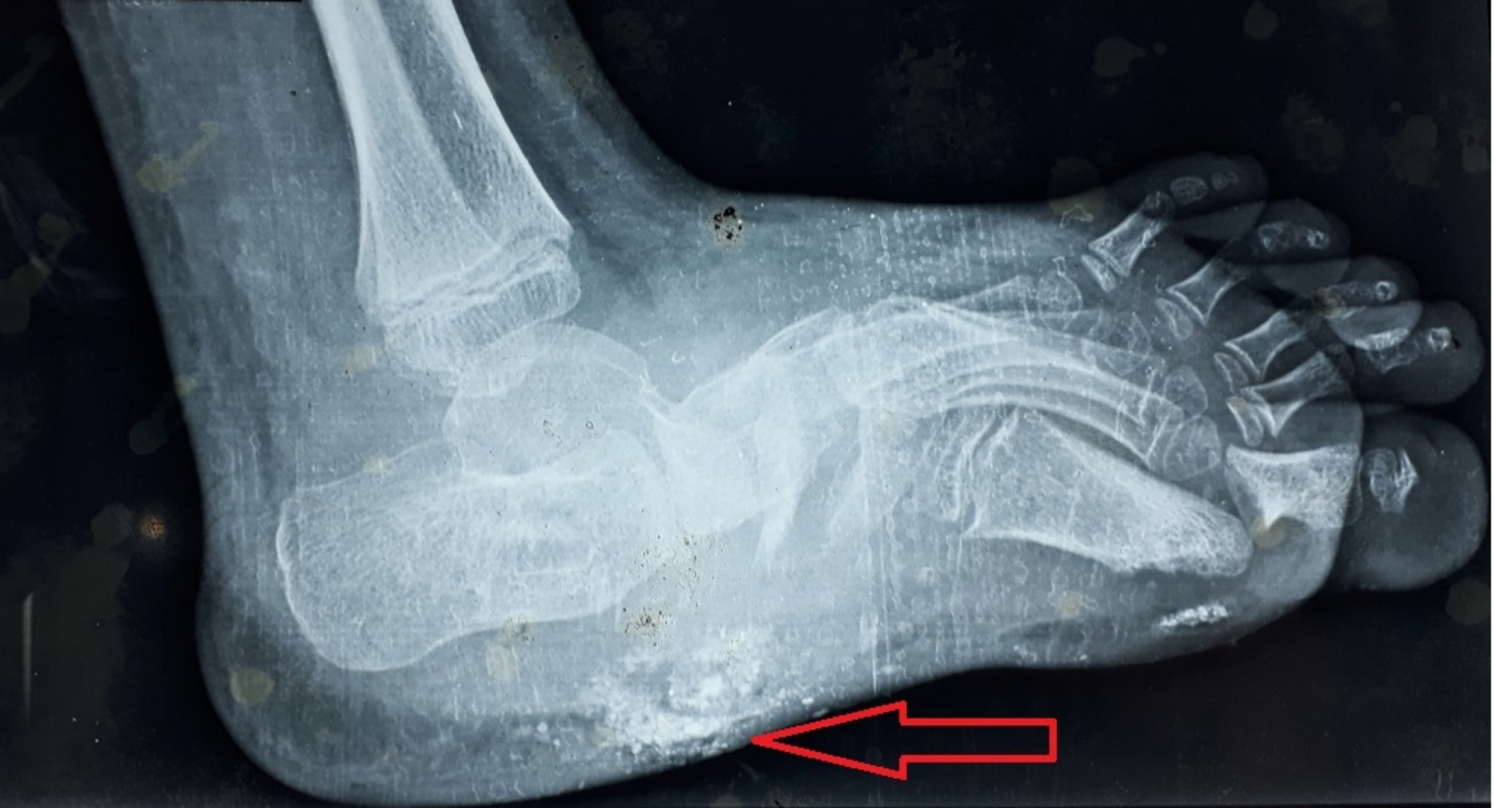
Magnetic resonance imaging (MRI) of the right foot showing lytic center with a ring of sclerosis in the tarsal bones

Electrophysiology tests, nerve conduction velocity (NCV) and electromyography (EMG), concluded the involvement of multiple lumbosacral roots on the right side and L4-L5-S1 roots on the left side. Multiple abscesses extending into deep tissues were revealed by ultrasound (US) scan of the right foot. On further investigation via a Doppler scan of the same foot, right dorsalis pedis artery (DPA) showed monophasic flow with normal peak systolic velocities which was suggestive of mild arterial insufficiency secondary to surrounding abscesses. Based on these findings, magnetic resonance imaging (MRI) of the right foot was ordered which demonstrated lytic center with a ring of sclerosis in tarsal bones (Figure 1). A triple-phase technetium 99 (Tc-99) based bone scan was done which showed increased uptake in all three phases, therefore, suggested OM involving tarsal bones of the right foot. Keeping in view the provided history, clinical presentation, lab results and the radiologic findings of this patient, a diagnosis of OM secondary to PM for the treatment of CF was made.

**Figure 2:**
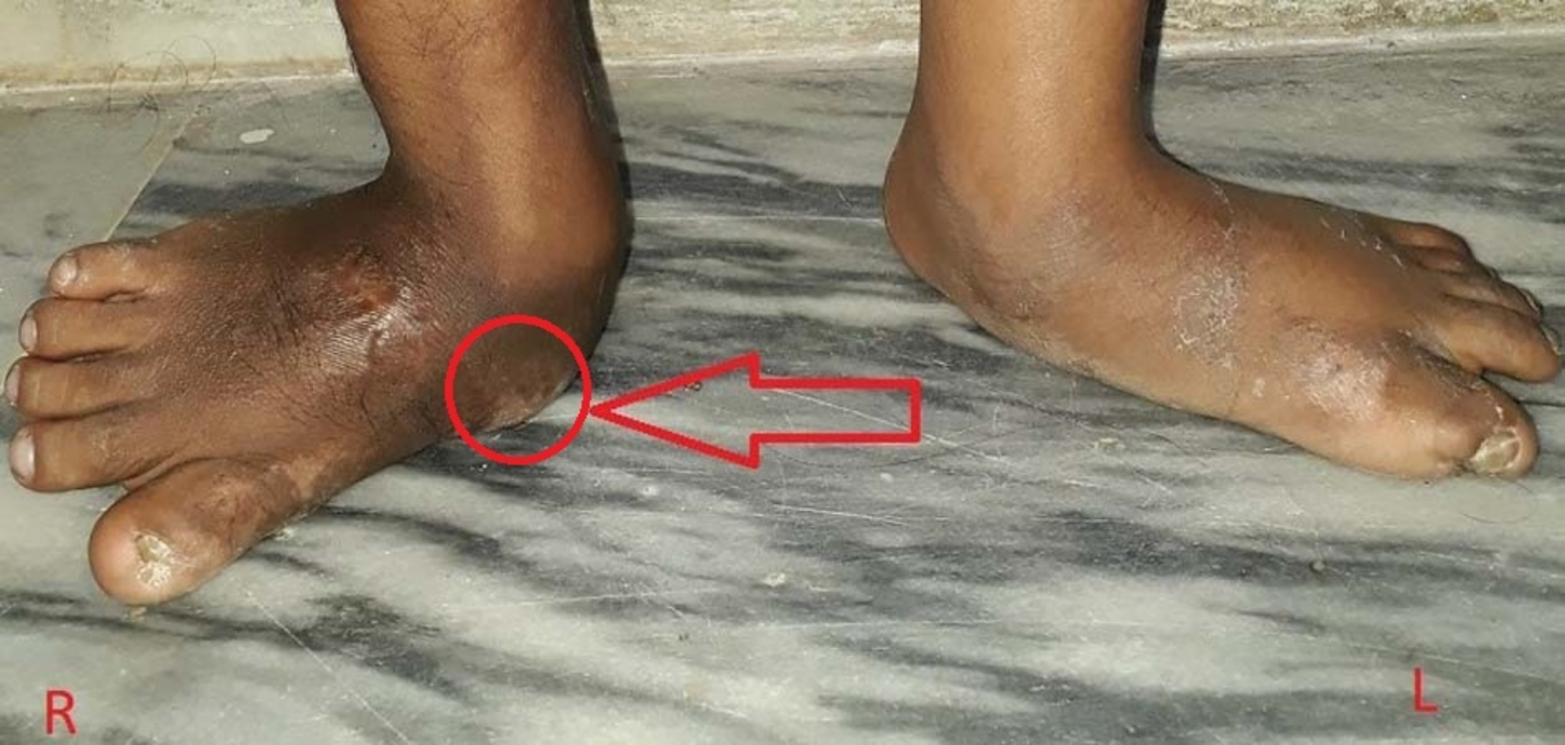
Healed ulcer in the right foot leaving behind scarred skin

The patient was initially started with intravenous (IV) ceftriaxone 900 mg 12-hourly along with vancomycin 360 mg 8-hourly. IV administration of provasc and dexamethasone were also given to relieve pain. Pus drainage was also done. After two weeks, ceftriaxone was replaced by IV ciprofloxacin 180 mg 12-hourly with the same dose of vancomycin. After another two weeks, ciprofloxacin was replaced by IV meropenem 360 mg 8-hourly again with the same dose of vancomycin. This regimen was also given for two weeks. The patient clinically subsided foot swelling and fever. Locally, the ulcer also healed leaving scarred skin (Figure 2). He ended up with the monotherapy of meropenem 360 mg 8-hourly as a prophylaxis to complete the antimicrobial course.

## Discussion

The PM was developed by Dr. Ignacio Vincent Ponseti in Iowa University in the 1950s for the correction of CF deformity which later proved to become a gold standard treatment option for idiopathic [7] or CTEV. Nevertheless, in cases of CTEV associated with MMC, a high correction rate of 94% has proven PM an effective option for non-idiopathic cases as well [8]. In contrast to the early preferred age of one month for the initiation of PM, our patient had a noticeable delay, starting the therapy at four years of age. However, Verma et al. reported an initial success rate of 90% in patients beginning treatment between one to three years [9]. The failure of PM in our scenario was largely due to non-compliance by parents and the obvious late age for the commencement of treatment. 

CTEV due to MMC differs from idiopathic CF because of its increased frequency of relapse, firmness, severe rigidity and greater complications [3]. Unfortunately, there is limited data discussing the adverse outcomes of CTEV or the long-term results of PM in non-idiopathic CF. A recent study in Thailand observed the rate of complications associated with ponseti serial casting which was found to be 17.8% with the major complication being cast loosening in 5.4% of the cases [5]. However, the incidence of infection was found to be a meagre 2.73% [5] making OM a rare and unique complication of casting as presented in our case. Gerlach et al., in his study, reported skin blisters in 32% feet by using foot abduction orthoses method [4]. However, none of them penetrated deep enough to cause infection.

A minor foot cellulitis following removal of the first cast, in this case, went unaddressed at the local clinical setup and shortly, a second cast was reapplied which later revealed skin abscesses. The untreated skin lesions lead to the contiguous spread of wound to the right tarsal bones resulting in exogenous OM. The sensory disorder in our patient due to MMC could be another contributory factor to the more serious complication as the ulcer did not come under clinical consideration. In some cases, the cascade of complications begins after contact dermatitis, another adverse effect of serial casting, the incidence of which was found in a Turkish study to be merely 3 out of 40 CF [10]. 

Diagnosis of OM starts from a thorough history and physical exam. The initial symptoms are usually fever with chills, local swelling, redness, and myalgias [11] as seen in our patient, thereby aiding in timely diagnosis. The pattern of fever, however, differed in this case in not being associated with chills. Physical examination for OM includes checking for neuropathy manifested by increased dorsiflexion with the knee flexed [11] as was found to be present in our patient.

CRP is highly sensitive and diagnostic for OM [11] and was substantially increased in the presented case. Usually, after one week of disease resolution, the value of CRP gradually returns to baseline. A raised level of TLC is another finding suggestive of OM as observed in this scenario. MRI, which is the investigation of choice for diagnosis and treatment was performed to confirm the diagnosis. An increased uptake in all three phases of Tc-99 bone scan further confirmed our diagnosis.

The treatment protocol for OM includes assessment of the patient's physiological and neurological status along with the degree of invasion of infection. Broad spectrum empiric antibiotics initiated followed by a more specific drug after the availability of culture and sensitivity data has been proved efficacious in the suppression of infection [11]. Our patient was started on third-generation cephalosporin along with vancomycin and the course of treatment was terminated using antibiotics from carbapenem family, bringing out successful rectification of OM.

## Conclusions

Keeping in mind all the aforementioned clinical findings and the predisposing factors to this rare complication of PM, it should be made necessary to perform a comprehensive physical exam and look for the signs of sensory deficits, especially in the lower limbs of patients with a history of MMC prior to initiating therapy. Moreover, after the application of each cast, hygiene should be maintained as the lower limbs, particularly the peripheries of older children, are often in contact with the floor during daily routine activities. Furthermore, the person applying a new cast must look for the presence of any contact dermatitis, cellulitis, abscess formation or any visible signs of infection, and if found, should be promptly addressed. Further research, elaborating the incidence of these deadly infections as a complication of CF treatment, should be considered so that other strategies can be planned for patients with sensory deficits.
